# Family perspectives and experiences on implementing telehealth in pediatric palliative care: a qualitative approach

**DOI:** 10.1007/s00431-025-06124-6

**Published:** 2025-04-08

**Authors:** Lucia Peñarrubia-San-Florencio, Carlos Godoy Junior, Silvia Ricart, Sergi Navarro Vilarrubí, Cristina Ruiz-Herguido, Arnau Valls Esteve, Julia Meca-Santamaria, Joan Blanco-Blanco, Laura Lahuerta Valls

**Affiliations:** 1 Pediatric Palliative Care Service, Sant Joan de Déu Hospital, Esplugues de Llobregat, Barcelona, Spain; 2 Institut de Recerca Sant Joan de Déu (IRSJD), Esplugues de Llobregat, Barcelona, Spain; 3 Faculty of Nursing and Physiotherapy and GESEC, Universitat de Lleida, Lleida, Spain; 4 Erasmus School of Health Policy and Management, Erasmus University Rotterdam, Rotterdam, Netherlands; 5 Sant Joan de Déu Foundation, Esplugues de Llobregat, Barcelona, Spain; 6 Innovation Department and Innovation, Health Technologies Group Sant Joan de Déu Hospital, Esplugues de Llobregat, Barcelona, Spain; 7 CIBER, Frailty and Healthy Aging (CIBERFES), Madrid, Spain; 8 Institute of Biomedical Research, GRECS, Lleida, Spain; 9 Granollers General Hospital, Granollers, Barcelona, Spain

**Keywords:** Pediatrics, Palliative medicine, Patient-centered care, Telemedicine, Caregivers

## Abstract

Telehealth is seen as a promising avenue to improve accessibility and quality of pediatric palliative care (PPC) yet engaging users with digital tools (DT) over the long term poses a challenge. Aligning telehealth initiatives with the actual needs and expectations of families is crucial for sustainable care models. To explore family perspectives and experiences with telehealth in PPC, focusing on their needs, perspectives, concerns, and hopes to refine digital care models. This research employed a qualitative methodology, gathering data through a combination of semi-structured interviews and focus group discussions with primary caregivers (PCGs) of PPC patients. PCGs of children utilizing telehealth services in PPC contexts. Interviews with seven individuals and a focus group of eight revealed three main themes: the experience of engaging with a PPC service that utilizes telehealth, the transformative potential of telehealth within PPC, and the barriers and facilitators influencing its adoption. A key finding was the collective desire among PCGs for telehealth solutions that support a patient and family-centered, holistic care model that integrates various health services without compromising the humanized essence of care. *Conclusions*: Families are generally positive about integration of telehealth into PPC, emphasizing the need for telehealth models that maintain the core values of humanized care. Success depends on engaging end-users throughout development, implementing user-friendly technology that fits into family life, and focusing on customization for patients.

**Table:** 

**What is known:** •* Telehealth has emerged as an innovative response to meet the increasing demand for highly specialized care in PPC.* • *Digital health solutions often face increasing abandonment rates over time.* **What is new:** • *Families caring for children with palliative needs are receptive to telehealth, identifying essential features for its long-term success.* • *They value the preservation of personal attention in care, emphasizing the importance of maintaining human connection alongside digital innovations.*

**Supplementary Information:**

The online version contains supplementary material available at 10.1007/s00431-025-06124-6.

## Introduction

Pediatric palliative care (PPC), as defined by the World Health Organization (WHO), is a medical specialty that delivers comprehensive care encompassing physical, psychological, social, and spiritual aspects to pediatric patients affected by life-threatening and life-limiting conditions and their families, with the goal of enhancing their quality of life [1]. Initiated at diagnosis, this continuous care transcends the patient’s therapeutic journey and can be delivered across settings, from hospitals to homes, ensuring personalized care that prioritizes the child’s comfort in their most familiar surroundings [2].

As the prevalence of chronic and complex conditions increases, the demand for PPC intensifies, presenting a considerable challenge for healthcare providers to offer high-quality, tailored care to patients and their families [3, 4]. It is estimated that each year, over 21 million children worldwide have special needs that require pediatric palliative care PPC [5]. The scarcity of specialized services equipped to deliver all-encompassing care to children with life-threatening and life-limiting conditions, especially those in home settings who depend on medical technologies, adds layers of complexity to care provision [6]. Research underscores the necessity of a holistic and multifaceted approach to home care for advanced-stage diseases, one that upholds rigorous safety standards irrespective of the setting chosen by the patient’s family [7, 8].

Information and communication technologies are now key in advancing healthcare quality, modernization, and sustainability [9]. Recognizing their value, WHO supports the incorporation of these technologies into homecare systems, citing the efficacy of hybrid models that integrate traditional and remote care methods. These models have not only expanded healthcare capabilities but have also yielded remarkable efficiencies [10, 11]. Although telehealth initiatives have predominantly catered to adults with chronic conditions that require consistent monitoring (chronic obstructive pulmonary disease, diabetes, heart diseases, or obesity) [12], they are now increasingly being adopted in palliative care (PC) settings. This shift towards including video consultations has expanded PC’s capacity, a development significantly hastened by the exigencies of the COVID- 19 pandemic [13].

Despite showing promising outcomes in various medical domains, the widespread adoption and effectiveness of telehealth programs within PPC still present uncertainties [14]. Moreover, trends indicate a potential decline in user engagement and motivation over time concerning the continuous use of digital health services [15]. In response to these challenges, there is a growing emphasis on designing digital health tools that are centered around the user’s needs and preferences. This study, conducted within the framework of the European AICCELERATE project [16], an initiative aimed at promoting the development of artificial intelligence and digital tools (DT) in healthcare, employs a qualitative methodology to delve into the perspectives, needs, and experiences of patients and primary caregivers (PCGs) engaged with a hybrid telehealth care model. The goal is to circumvent the common pitfall of creating sophisticated technologies that fail to address the real-world needs of their target demographic, thereby enhancing the success rate of technology adoption post-implementation.

## Study objectives and purpose

### Primary objective

Explore families’ perceptions of using telehealth for PPC, aiming to understand their needs, perceptions, concerns, and expectations. This contributes to enhancing telehealth care models in PPC settings.

### Secondary objectives

To investigate families’ views on integrating telehealth solutions into PPC, identifying perceived benefits and challenges.

To analyze key features families, deem essential for the long-term viability of tele-assistance in home settings, thereby promoting the effective development and implementation of user-centered telehealth interventions.

## Methodology

### Study design

This study employed a qualitative approach, incorporating semi-structured interviews, focus groups, and field notes from the researcher to address the research objectives.

### Data quality criteria

The methods and reports adhered to the Consolidated Criteria for Reporting Qualitative Research (COREQ) checklist [17].

### Ethical considerations

Adhering to the Helsinki Declaration and Human Medical Research Law, the study received approval from the local ethics committee of Hospital Sant Joan de Déu (PIC- 123–19). Informed consent was obtained from all participants prior to their inclusion, providing them detailed information about the study’s objectives and procedures. Participation was entirely voluntary, with no material incentives offered.

### Population and sample

Participants were primary PCGs of children with life-threatening and life-limiting conditions, receiving care under the telehealth hybrid model of the PPC unit at Hospital Sant Joan de Déu, Barcelona. Since 2021, this PPC unit has integrated telehealth into its services, including telemedicine and digital platforms for information and communication, such as a patient portal [18]. Eligibility for inclusion required individuals to have been PCGs of a child under the PPC unit’s supervision for at least 6 months prior to joining the study. Exclusion criteria included PCGs of children in their final days of life and language barriers (limited proficiency in Spanish or Catalan).

We employed purposive sampling to ensure a diverse and heterogeneous participant base. Recruitment was led by the principal investigator (LPS, PhD candidate). Recruitment involved detailed study explanations over the phone, sending informed consent documents through the institutional portal, and collecting signed consent during a physical visit with the research team.

### Data collection

Data collection utilized a blend of in-depth individual interviews, focus groups, and entries from a researcher’s field journal capturing observations and reflective insights [19]. Both the semi-structured interviews and the focus groups were conducted remotely, with participants joining from their homes via smartphones, tablets, or personal computers. The data collection period spanned from January to April 2022. The semi-structured interviews and focus groups were led by the principal investigator (LPS) and a member of the associated research team (LLV, PhD). Both women researchers bring together over two decades of experience in health services research. The principal investigator, an expert PC nurse, was familiar to the participants, although not serving as their direct care nurse.

An interview guide based on literature and the research group’s experience was developed (Supplementary Material 1), covering topics on the conception and use of new technologies in PPC. Additionally, open-ended questions were included to explore how new information and communication technologies could enhance the care and well-being of the child and family (Supplementary Material 2). Concurrently, a member of the associated research team kept a field journal to record observations during the interviews and focus group (SRV, PhD).

Interviews were recorded in audio and video, then transcribed, and participants’ names were anonymized by the principal investigator. After transcription, the audio and video recordings were deleted.

### Data analysis

The qualitative analysis adopted an inductive approach, enabling the identification of emerging patterns and the discovery of insightful concepts within the complexity of pediatric palliative experiences [20]. Coding and analysis were performed in pairs, parallel to data collection, including a team member (CGJ, PhD candidate) not directly engaged in conducting interviews or focus groups [21]. A coding framework was developed, modified, and refined using ATLAS.ti 22 software as new themes and subthemes emerged throughout the analysis (Supplementary Material 3). Two discussion meetings were held among the reviewers to aid in the interpretation of data and to validate the emerging themes identified.

The total number of interviews was determined by reaching data saturation. Following this, a focus group was conducted to specifically delve into the emerging categories from the semi-structured interviews and triangulate the collected information.

## Results

We conducted seven semi-structured individual interviews. To further validate and refine our findings, a focus group session was held with eight new participants, distinct from those in the initial interviews. This step facilitated the triangulation of data and the fine-tuning of previously identified categories. There were no declines to participate among those contacted. The average duration of the interviews was 31 min, with a range of 24 to 43 min, while the focus group session extended to 75 min.

All participants were in a private setting during their interview, except for one mother, who was accompanied by her breastfeeding infant, and a pair of caregiver participants who joined the interview together. Table 1 outlines the sociodemographic details of both the semi-structured interview and focus group participants, including information about the patients under their care.

**Table 1:** Demographic and clinical characteristics of caregivers and patients included in the study

Characteristics	Number (*N*)	Percentages (%)
**Caregivers’ gender**		
Male	*7*	*47*
Female	*8*	*53*
**Caregivers’ age**		
< 30	*3*	*20*
30–40	*7*	*47*
> 40	*5*	*33*
**Marital status**		
Married or cohabiting	*14*	*93*
Divorced or not cohabiting	*1*	*7*
**Patients’ underlying illnesses**		
Oncological	*3*	*20*
Neurological	*8*	*53*
Other	*4*	*27*
**Patients’ age**		
< 1	*2*	*13*
1–5	*5*	*33*
6–12	*3*	*20*
13–16	*4*	*27*
> 16	*1*	*7*
**Patients’ gender**		
Male	*9*	*60*
Female	*6*	*40*
**Siblings**		
0	*6*	*40*
1	*6*	*40*
2	*3*	*20*
3 or +	*0*	*0*
**Medical devices at home**		
0	*3*	*20*
1	*3*	*20*
2	*4*	*27*
3 or +	*5*	*33*

After the iterative analysis of the data, 12 codes were consolidated from the original 76 codes, which were grouped into 3 main categories after axial coding. Table 2 shows the main themes and subthemes derived from the analysis.

**Table 2:** List of main themes and sub-themes from the analysis

Themes	Sub-themes
C.1. Insights into hybrid care: families’ experiences with the support received from the current hybrid PPC	C.1.1. Direct interaction with individuals and personalized attention
	C.1.2. Fluid communication and empowerment
	C.1.3. Restricted availability and discontinuity in care
C.2. Shaping the future: leveraging information and communication technologies to transform PPC	C.2.1. Enhancement of communication and information
	C.2.2. Coordination and integration among community health services (care network)
	C.2.3. Modernizing care approaches and enhancing family empowerment
	C.2.4. Comprehensive and holistic support
C.3. Advancing PPC through digital transformation: barriers and facilitators	C.3.1. Maintenance of human connections
	C.3.2. Security and privacy
	C.3.3 Initial instruction and ease of use
	C.3.4 Diligent use
	C.3.5 Preference for mobile devices

### Insights into hybrid care: families’ experiences with the support received from the current hybrid PPC

This category encapsulates the experiences, needs, and evaluations concerning the support patients and their families receive from the hybrid service provided by the PPC team. Central to the service provided by the hybrid telehealth PPC is the provision of humanized care, a fundamental need highlighted by all participants as a defining characteristic of the current care approach. Key elements of this humanized care include direct contact, personalized support tailored to the unique circumstances, and progression of the patient’s condition (Q#1, Q#2); empowerment of PCGs through ongoing education offered by healthcare professionals; and clear, fluid communication with the clinical team (Q#3).

*Q#1—*“…hearing your voices is what relaxes us…”

*Q#2—*“…the peace of mind that you [PPC providers] can see it, if something [wrong] is happening…”

*Q#3—*“Seeing you [PPC providers] and speaking with a professional, I think, gives you [the PCGs] more confidence, more capability.”

A significant concern among participants was the restricted availability of the specialized PPC team, noting that access to these expert services is not always possible around the clock. This limitation poses considerable challenges, especially when PCGs encounter new, acute, or unexpected health crises. In such situations, the most commonly used alternative is to seek help from local emergency services, which may not be adequately equipped to address the specialized needs of PPC patients effectively (Q#4). This discontinuity in care exacerbates PCGs’ uncertainty and distress. In the absence of immediate access to PPC specialists, some PCGs opt to travel to the hospital where the PPC team is based. However, this decision presents its own set logistical of challenges, including travel distances and the added strain of managing transportation (Q#5).

*Q#4—*“When you visit [the emergency service] for a respiratory issue, the immediate response in primary care emergencies is to administer salbutamol. However, in cases like my child’s, salbutamol is inappropriate because the underlying condition differs. This reflects a systemic issue where individual patient needs are overlooked in favor of a one-size-fits-all approach.”

*Q#5—*“The hospital is always crowded, and for me, it’s a hassle to get the child into the car, drive there, find parking, and it takes a long time to get to Barcelona depending on the time of day. If it’s something urgent, then I have no choice but to go…”

### Shaping the future: leveraging information and communication technologies to transform PPC

Participants engaged in envisioning scenarios and solutions for integrating digital technologies into the PPC service model. A significant number of participants showed enthusiasm for trying out new digital solutions, demonstrating an openness to adopting technological advances in PPC. They especially appreciated how these technologies could offer continuous insights into their children’s health status, thereby minimizing the need for hospital visits and enhancing overall communication (Q#6, Q#7).

*Q#6—*“Having the ability to receive updates on my child’s condition through virtual means or a simple phone call is incredibly helpful. It eliminates the need for in-person visits, which can be cumbersome…. for regular updates, this virtual approach is ideal and much appreciated.”

*Q#7—*“Sometimes we don’t really know how to interpret what the problem is. We can see trends, we can see things, but it’s a bit hard [identifying and understanding the specific health issues or needs of patients]. I think it would be very good if there was a direct transfer of information [real-time remote monitoring].”

Moreover, participants viewed DT beneficial for better care coordination among all parties involved in a child’s treatment (Q#8). Additionally, they highlighted the importance of providing parents with the necessary tools and information to manage the complexities of their child’s care more effectively, thereby fostering greater empowerment and ensuring that care practices remain up to date with the most current advancements and resources (Q#9). Reflecting the holistic approach of PPC, they also recognized the value of these technologies in delivering comprehensive support that spans beyond medical care, including emotional support (Q#10).

*Q#8—*“There needs to be very good communication between the home environment, the hospital environment, and the school environment, as children spend many hours at school. And the information, the ability to respond, all of this also needs to be present there.”

*Q#9—*“Parent training should focus on helping us become more independent and informed about new developments of any new or existing support available. We’re currently discovering resources we were previously unaware of, and this affects us all in various ways: logistics, family finances, and our child’s well-being.”

*Q#10—*“Emotional support is also crucial for parents.”

### Advancing PPC through digital transformation: barriers and facilitators

This category identified central elements for integrating digital solutions into PPC, aiming for a balanced approach between technology and traditional care. At the core of this integration lies the principle of the maintenance of the humanized care, highlighted by participants’ preference to keep personal interactions alive within digital care settings (Q#11). While some degree of automation is acceptable, participants emphasized that it should enhance rather than replace the human element and direct connection with the care team (Q#12). Additionally, PCGs expressed confidence in the care team’s ability to handle digital information securely, placing responsibility for data protection on healthcare providers (Q#13).

*Q#11—*“Sometimes virtual assistance might not be sufficient. Sometimes the help of a professional at home is inevitable.”

*Q#12—*“I feel more secure talking to a person…..”

*Q#13—*“We know where the data goes; it will be sent directly to you. I don’t think it can get lost in the network; it should be secure enough, I believe….”

The need for simplicity and smooth integration of digital technologies into daily routines was another key theme (Q#14). Participants expressed feeling overwhelmed by the demands of caregiving at home, highlighting the necessity for DT to alleviate rather than exacerbate these challenges (Q#15).

*Q#14—*“Provided I receive proper training, and the devices aren’t overly complicated to use, I’m completely comfortable with it.”

*Q#15—*“We have so much work every day that if it’s going to involve more work on our part [the use of DT], then no.”

When it comes to digital instruments for collecting patient data that might require caregiver involvement, opinions varied: some preferred tools that demanded minimal, sporadic engagement (Q#16), whereas others were open to more frequent interaction if it tangibly aided their children (Q#17). Additionally, a preference emerged for using mobile devices like smartphones or tablets for these digital interactions (Q#18).

*Q#16—*“Sending the data whenever we notice something that warrants attention is crucial.”

*Q#17—*“It wouldn’t feel like a burden at all [completing online questionnaires], primarily because it concerns my son. I’m confident I can dedicate at least 10 min daily to provide all the necessary data about him.”

*Q#18—*“I have a computer at home that’s mostly collecting dust; my phone is what I primarily use now, given the computer’s obsolescence. Essentially, the smartphone is my go-to device.”

## Discussion

The main objective of this study was to delve into families’ perspectives on the use of telehealth and digital solutions in the field of PPC, to understand their needs, perceptions, concerns, and expectations. Our findings reveal an interest among families of children with complex illnesses and PPC needs towards the integration of these technologies into daily care. They foresee a future where technology is integrated with traditional care, forming a harmonious synergy. This integration is seen as a means to improve parental participation in home care settings, thereby strengthening home care capabilities, increasing family autonomy, and streamlining the care process. Such a vision aligns with existing research highlighting the empowerment of families through the implementation of DT in PPC contexts [22].

Interestingly, our study participants displayed openness to adopting new digital innovations, without any discernible apprehension. This receptiveness to digital health solutions may be influenced by our sample’s demographic profile, mainly comprising individuals younger than 40. Previous studies have shown that this demographic, being more versed in digital technology, often possesses a high degree of digital fluency, essential for adapting to technological advancements [23]. Additionally, the experience gained from integrating various digital health solutions during the COVID- 19 pandemic has likely fostered their adaptability and comfort with these technologies [24]. Notably, our findings also reveal a lack of significant concerns over privacy among participants. They seemed at ease with the idea of sharing sensitive data digitally. This contrasts with the prevalent privacy concerns observed in adult PC settings, where the introduction of telehealth tools often leads to anxieties over data privacy and management [25].

A paradox emerged in our study. While DT are valued for enhancing communication with PPC teams, there is a simultaneous worry that they may dilute the crucial human connections between healthcare professionals and families. PCGs recognize that, although digital solutions streamline care processes, they cannot substitute the essential elements of trust, empathy, and personal connection that are particularly crucial during the difficult times of illness. This concern aligns with similar research findings [26–28], suggesting that telehealth, despite its benefits, cannot replicate the comprehensive depth and quality of face-to-face care. Echoing the sentiments of prior studies, the need for DT to blend effortlessly into family life was emphasized [25]. Participants advocated for user-friendly technologies that are straightforward to use, set up, and manage, considering the significant emotional and physical stresses families face. The challenge of configuring and handling these devices adds an extra layer of burden.

The study has some limitations that should be taken into account for the correct interpretation of the results. The participant sample of the study was limited to a single PPC service in Barcelona, Spain. This team integrates an asynchronous communication pathway with patients and families through the patient portal, meaning that participants were not entirely unfamiliar with adopting or assessing new technologies. Given that healthcare practices and levels of digital literacy can vary significantly between regions, the applicability of our findings might be limited beyond this specific context [29]. Expanding the study to include caregivers from other centers, particularly those without prior exposure to digital tools, could provide valuable insights and enhance the generalizability of the results. Future research could explore this broader inclusion to better assess digital adoption in more diverse care settings.

Furthermore, all interviews in our study were conducted online, either due to participants’ preference or logistical reasons. Although existing research suggests that both face-to-face and virtual interviews generate similar amounts of data and thematic richness, face-to-face interactions tend to be more profound [30]. Despite this, remote interviews via video, phone, or online platforms remain reliable and credible methods [31]. Finally, the interviews and focus groups were carried out without the use of a physical prototype or actual DT that might be implemented in PPC. This lack of tangible examples may have restricted participants’ grasp of certain practical applications, while encouraging speculation about various features and potentialities. Direct interaction with these tools could uncover unexpected benefits or obstacles, thereby altering users’ viewpoints and expectations [32].

## Conclusion

This study highlights the critical importance of addressing the needs of PCGs and children in the realm of hybrid PPC. It brings attention to family concerns that technological advancements could potentially detract from the quality of care by replacing vital human interactions or by adding to their existing burdens. Such insights underscore the critical need for a thoughtful approach in the ideation, design, and development of future digital solutions, with an aim to augment rather than replace face-to-face care, prioritizing personal connections, customized care, and user-friendly technology.

Moving forward, it becomes imperative to also incorporate the viewpoints of clinical staff, another key user group, to ensure that the development of any new tool effectively responds to the comprehensive needs of the entire end-user community before its design is finalized.

## Supplementary Information

Below is the link to the electronic supplementary material.

Supplementary file1 (DOCX 17 KB)

Supplementary file2 (DOCX 22 KB)

Supplementary file3 (XLSX 13 KB)

## Data Availability

No datasets were generated or analysed during the current study.

## Abbreviations

COREQ: Consolidated criteria for reporting qualitative research
DT: Digital tools
PC: Palliative care
PCGs: Primary caregivers
PPC: Pediatric palliative care
WHO: World Health Organization
