# Supplementary material for: Family perspectives and experiences on implementing telehealth in pediatric palliative care: a qualitative approach
Source: Eur J Pediatr. 2025 Apr 8;184(5):287. doi: 10.1007/s00431-025-06124-6 (PMC11978708; doi:10.1007/s00431-025-06124-6)
Supplement: Supplementary file 1 — Supplementary file1 (DOCX 17 KB) [file 431_2025_6124_MOESM1_ESM.docx]

**Semi-structured interview guide for caregivers (families)**

1. How do you use technology in your everyday life?
2. Tell us about how you use technology at home. What’s that like for you?
3. What does it mean for you to have to go to the hospital for visits?
4. Have you ever had a doctor’s appointment over video? Did it feel different from being there (in the hospital, in the clinic) in person?
5. Have you ever been involved in any projects to implement or pilot new digital tools in your child's healthcare environment? What kind of technology was it?
6. What are your thoughts on your healthcare team being able to monitor medical equipment you use at home, such as breathing aids or heart monitors, online to ensure everything is working well from afar?
7. What do you think about adding more technology into the way you currently receive care?
8. Do you have any concerns about the hospital or healthcare team using the information from these technologies? What worries you about this?
9. Thinking about the care you get right now, what are some ways you think it could be better?
10. What changes do you think are needed to make it possible to get more of your care through the computer or phone (virtual care)?
11. Is there anything else you’d like to talk about or any other thoughts you want to share?
