# Supplementary material for: Family perspectives and experiences on implementing telehealth in pediatric palliative care: a qualitative approach
Source: Eur J Pediatr. 2025 Apr 8;184(5):287. doi: 10.1007/s00431-025-06124-6 (PMC11978708; doi:10.1007/s00431-025-06124-6)
Supplement: Supplementary file 2 — Supplementary file2 (DOCX 22 KB) [file 431_2025_6124_MOESM2_ESM.docx]

**Reflective Question Set**

**Support for Question 1:**

- What resources or devices do you use daily?
- Have you made any adaptations in your home to incorporate new technologies to support your child’s independence (like using voice assistants, fingerprint scanners, iPads for communication, etc.)?
- Regarding information and communication:
- Do you use official/institutional apps or servers to communicate with healthcare services (patient portals, etc.)?
- How do you typically access health information or content?

**Support for Question 2:**

- What advantages have you noticed with the use of these technologies?
- What are some disadvantages or inconveniences you've encountered?

**Support for Question 3**:

- For example, considering the logistics and cost of traveling, and work implications:
- After hospital visits, considering all the discussed resources, do they usually meet your expectations (in terms of resolving doubts, addressing issues, patient assessment, explanation of the follow-up plan), or do you think it could have been done virtually?

**Support for Question 4:**

- Do these phone consultations meet your and your child's needs as effectively?
- Can you compare the experience of phone or video call consultations?

**Support for Question 5:**

- If yes, please describe the context.
- How do you feel about participating in this?
- What are your feelings about security, connection with the hospital/medical team, time spent, etc.?

**Support for Question 6:**

- What would you like us to monitor remotely?

**Support for Question 7:**

- What would convince you to accept this proposal?
- What concerns might make you reject or be uncertain about this proposal?
- Do you think it could be an invasive tool regarding your privacy/child’s care?
- Could you feel sidelined or judged as the main caregiver?
- Do you believe this could improve the care received or is it just another way to provide care?
- Who do you think should initiate the conversation with the digital tool (family or professional)?
- If the family initiates contact due to concerns during off-hours/at night, would you prefer direct contact with a professional or through a future tool?
- Routine and daily question protocols may be required for information entry. To make it practical for daily life, how many questions do you think you could answer per day or week? What level of interaction (dialogue with the tool/need to input parameters) do you find manageable with your daily routine?

**Support for Question 8:**

- Do you think this information might be overlooked more than when you report directly to professionals?
- Do you have concerns about privacy invasion?
- What are your thoughts on the device having a camera controlled by the hospital?
- Are you concerned that this information could be accessible to others?
- Would you be comfortable if there isn’t real-time feedback from telemonitoring?
- Would you be at ease if the caregiver has to input the data?
- Would you prefer to make adjustments to the remotely monitored devices yourself, or would you rather the healthcare team make remote adjustments?
